# Natural Killer Cell Immunotherapy: From Bench to Bedside

**DOI:** 10.3389/fimmu.2015.00264

**Published:** 2015-06-03

**Authors:** Anna Domogala, J. Alejandro Madrigal, Aurore Saudemont

**Affiliations:** ^1^Anthony Nolan Research Institute, London, UK; ^2^University College London, London, UK

**Keywords:** natural killer cells, cancer, immunotherapy, activation, proliferation, persistence

## Abstract

The potential of natural killer (NK) cells to target numerous malignancies *in vitro* has been well documented; however, only limited success has been seen in the clinic. Although NK cells prove non-toxic and safe regardless of the cell numbers injected, there is often little persistence and expansion observed in a patient, which is vital for mounting an effective cellular response. NK cells can be isolated directly from peripheral blood, umbilical cord blood, or bone marrow, expanded *in vitro* using cytokines or differentiated *in vitro* from hematopoietic stem cells. Drugs that support NK cell function such as lenalidomide and bortezomib have also been studied in the clinic, however, the optimum combination, which can vary among different malignancies, is yet to be identified. NK cell proliferation, persistence, and function can further be improved by various activation techniques such as priming and cytokine addition though whether stimulation pre- or post-injection is more favorable is another obstacle to be tackled. Here, we review the various methods of obtaining and activating NK cells for use in the clinic while considering the ideal product and drug complement for the most successful cellular therapy.

## Introduction

Natural killer (NK) cells are unique lymphocytes, distinct from B and T cells, which bridge the innate and adaptive immune systems. They have the unique capacity to exert immunoregulatory and cytotoxic functions against transformed and infected cells without prior sensitization. NK cells are characterized by the expression of CD56 and absence of CD3 and can be further subdivided into a CD56^bright^ population, which is predominantly cytokine producing and a CD56^dim^ population, which is cytolytic and provides antibody-dependent cell-mediated cytotoxicity via CD16 (1). NK cells operate by detecting information, which is missing on the target. This phenomenon is known as the “missing self hypothesis” and postulates that NK cell cytotoxicity inversely correlates with the target expression of major histocompatibility complex class I (MHC-I) (2, 3). In addition, NK cell activity is further regulated by a complex array of inhibitory and activating receptors such as killer cell immunoglobulin-like receptors (KIR), natural cytotoxicity receptors (NKp44, NKp30, and NKp46), and C-type lectins (CD94/NKG2A/NKG2C/NKG2D). These properties equip NK cells with the tools to actively eliminate susceptible targets (4). Taking into account, the cytotoxic potential of these cells numerous attempts have been made to transfer NK cell immunotherapy into the clinic. Here, we review which methods to consider for obtaining cells for therapy, drug complements, and pre-infusion activation techniques. We also summarize current clinical trials and outcomes and postulate where success in NK immunotherapy may lie.

## NK Cells and Cancer

Natural killer cells were first implicated as playing a role in cancer immunosurveillance when one large epidemiologic study found that low NK cell cytotoxicity forecasted an increased risk in developing cancer (5). There have since been numerous studies, which demonstrate that NK cells can target human tumors *in vivo* making them a desirable candidate for therapeutic use (6). Clinical trials using autologous NK cells have shown the therapy to be non-toxic, however, they fail to prove efficacy (7), which could be the result of inhibition by self-MHC-I. Allogeneic treatment therefore has potential to offer an alternative therapy with improved effect. The direct involvement of allo-reactive NK cells in inducing anti-tumor effect in hematopoietic transplants was first demonstrated in 2002 (8). NK cells showed to enhance engraftment; providing graft vs. leukemia (GvL) effect while suppressing graft vs. host disease (GvHD) particularly when a KIR ligand mismatch in the donor to host direction was observed. Reduced GvHD was hypothesized to be attributed to the lysis of the recipient’s antigen presenting cells (APCs) reducing the incidence of GvHD while maintaining GvL effect. This was later successfully translated into an *in vivo* model using acute myeloid leukemia (AML)-engrafted NOD/SCID mice infused with allo-reactive NK cells. Tumor clearance was achieved implicating NK cells in preserving the GvL effect (9).

Miller and colleagues later translated NK cell therapy alone into the clinic where allogeneic NK cells were infused into patients with advanced cancer alongside IL-2 administration. This demonstrated that NK cell infusions were feasible and safe and led to complete remission in 5/19 patients with poor prognosis AML (10). Additionally, the efficacy of haploidentical NK cell therapy in the refractory disease was further improved by depleting host regulatory T cells with IL-2 diphtheria toxin preventing their immunosuppressive effect (11). NK cell allo-reactivity could also be utilized in other scenarios besides hematopoietic stem cell transplantation (HSCT) with studies in malignant glioma and neuroblastoma patients demonstrating that NK cell infusions are safe and partially effective (12, 13). Numerous types of cancer could therefore benefit from NK cell immunotherapy and current clinical trials include pancreas, lungs, head/neck, breast, and renal cell carcinomas.

## Clinical Conditioning

Not only chemotherapy and/or radiotherapy are required for the success of HSCT but also cellular immunotherapy. Such treatments are necessary to reduce tumor burden and suppress the immune system of the patient to prevent rejection of the cellular therapy. Defining the correct conditioning regimen is therefore critical. In a transplantation setting, common regimens are referred to as myeloablative, non-myeloablative, and reduced intensity and their use will depend on patient age and disease severity; however, any decrease of leukemia recurrence is often at the expense of an increase in toxicity (14).

The use of new conditioning agents termed as “novel agents” have become increasing popular in cancer immunotherapy as a result of their immunomodulatory and direct tumor targeting mechanisms. In combination with cellular therapy, they offer the potential for a more personalized and less toxic treatment regimen as these specialized drugs have been shown to not only reduce tumor burden but also enhance the function of cellular therapies. Although chemotherapy has revolutionized the treatment of cancer, its side effects include the development of refractory disease and severe toxicity. Novel agents provide an alternative option of harnessing the immune system to tackle malignancies.

Thalidomide was one of the first novel agents to be well studied; it is a synthetic glutamic acid derivative that is capable of immunomodulatory, anti-inflammatory, and anti-angiogenic effects. Although proven successful in targeting multiple myeloma the exact mechanism of action of thalidomide is yet to be elucidated although anti-inflammatory effects have been attributed to inhibition of TNF-α production by monocytes and anti-proliferative capabilities to disruption of the bone marrow (BM) microenvironment preventing multiple myeloma cellular development (15). Although extended anti-angiogenic characteristics make a desirable option in limiting tumor development its immunomodulatory properties have not been so well defined. Lenalidomide is an immunomodulatory compound with a dual mechanism of action. It is capable of targeting the tumor directly through stromal support disruption, induction of tumor suppressor genes, and activation of caspases (16). It is also able to stimulate the cytotoxic functions of NK cells and T lymphocytes while limiting the immunosuppressive impact of regulatory T cells (17). Additionally, bortezomib is a proteasome inhibitor proven popular by up-regulating expression of TRAIL death receptors and altering caspase-8 activity rendering tumors susceptible to NK cell lysis. However, intriguingly these tumors became resistance to T cell cytotoxicity (18). The specific mechanisms by which novel agent function offer a promising future for the treatment of a variety of malignancies as these agents target not only the tumor themselves but also offer potential to enhance the immune system. This provides the possibility of coupling cellular therapy with novel agents to provide personalized treatment regimens to target an individual’s condition.

## NK Cell Sources

It is considered that the success of NK cell immunotherapy is dependent on obtaining high numbers of functional NK cells that have the potential to survive *in vivo*. Numerous attempts have therefore been made to obtain high levels of NK cells from a variety of sources. One option is to isolate cells directly from peripheral blood (PB) or cord blood (CB), however, as NK cells make up only 10% of circulating lymphocytes in PB and 20% in CB the number of cells obtained can be limited and could potentially prevent the option for multiple infusions. Doses of 1–2 × 10^7^ cells/kg have been identified as safe (19); however, higher doses of 2 × 10^8^/kg have been shown to be well tolerated and non-toxic (20). Several techniques have therefore been explored to increase cell numbers. This includes expanding isolated cells *in vitro* using different combinations of cytokines with or without feeder layers, the use of NK cell lines and differentiating NK cells from hematopoietic stem cells (HSCs).

## NK Cell Expansion

Numerous methods of expanding mature NK cells *in vitro* have been explored and have been reviewed previously (21); however, these products seem to produce limited clinical success. This may be because of wide variations in expansion rate and distribution of NK cell subpopulations (22) or expanding mature cells produces effectors with a more finite lifespan unable to proliferate with lower cytotoxicity post infusion (23). NK cell expansion using aAPCs particularly the GMP-compatible genetically modified form of the K562 myeloid leukemia cell line engineered to express membrane-bound interleukin 15 and the ligand for the co-stimulatory molecule 4-1BBL has been rising in popularity due to the potential to rapidly expand an NK cell product with an up-regulation of activating receptors and improved killing capacity (24). However, a first-in-human trial carried out by Shah and colleagues in 2014 performing the adoptive transfer of donor-derived IL-15/4-1BBL activated NK cells showed interesting results. Surprisingly, 5/9 patients experienced acute GvHD and as the T cell content of the infusion was well below the specified threshold for GvHD development the group concluded that the aNK-DLI contributed to the effect by stimulating underlying T cell allo-reactivity (25). This is the first time in a clinical setting NK cells have been implicated in the role of induction or aggravation of GvHD, which could be a result of lack of immunosuppressive drugs post transplant or infusion of IL-2, which expands immunoregulatory populations. This coupled with the infusion of an expanded NK cell population with such a high up-regulation of activating receptors could be the reason for such unfavorable results.

## NK Cell Lines

The use of NK cell lines have been seen as an attractive option due to the availability of a clinical grade frozen stock and their homologous nature. The most prominent NK cell line currently in focus is NK-92, which was established from a patient with non-Hodgkin’s lymphoma and has demonstrated the capability of lysing leukemia, lymphoma, and myeloma *in vitro* (26). Current clinical trials have proven non-toxic; however, they have shown limited success in demonstrating efficacy (27, 28). This could be a result of the necessity to irradiate a cell line prior to infusion for safety requirements, the cells could therefore be incapable of proliferation *in vivo* severely limiting their persistence and potential to target the tumor.

## Differentiation of NK Cells

Differentiating NK cells *in vitro* from HSCs or induced pluripotent stem (iPS) cells are alternative options for obtaining high numbers of functional cells. Different sources of cryopreserved HSCs have been used to differentiate NK cells *in vitro* including human embryonic stem cells (hESC), BM, mobilized peripheral blood stem cells (mPBSC), and cord blood stem cells (CBSC). hESC are a controversial source due to the ethical dilemma posed by obtaining cells from a 5- to 7-day-old embryo. However, the H9 hESC cell line has been used to produce NK cells that express activating and inhibitory receptors, including KIRs, and are able to produce cytokines and mediate cytotoxicity *in vitro* and *in vivo* (29). The invasive collection procedure limits the use of BM and has therefore mainly been used to study NK cell development (30, 31). Differentiating NK cells from induced pluripotent cells offers potential due to the ready availability of a donor and the non-invasive cell harvesting methods. A recent study identified a method of differentiating mature and functional NK cells using a combination of embryoid body formation and membrane-bound interleukin 21-expressing aAPCs (32) and a thorough review of the potential uses of such cells in the clinic was published last year (33). The possibility of reprograming cells is a promising one; however, there is the possible limitation that the differentiated NK cells will be suppressed by self-MHC and therefore have little cytotoxic effect. The use of NK cells differentiated from CD34^+^ progenitors was first shown to be feasible in the clinic by Yoon and colleagues in 2010 (34). This led to interest in the use of umbilical cord blood CD34^+^ cells as a source of NK cells with the focus being on generating a readily available, non-invasive, off the shelf cellular product (35). Our group modified a published protocol (36) and compared the use of mPBSC, fresh CBSC, and frozen CBSC at differentiating NK cells *in vitro* (37). This work demonstrated frozen CB CD34^+^ cells to be the best source of NK cells over fresh CB CD34^+^ and frozen mPBSCs. This was due to higher fold expansion and therefore higher NK cell numbers generated without compromising on phenotype, cytokine production, or cytotoxicity. Additionally, the cells are capable of further proliferation *in vitro* and more importantly could persist for longer and in higher numbers *in vivo*. Considering that proliferation and persistence of NK cells *in vivo* is fundamental for the development of a clinically relevant cellular product this makes the differentiation of NK cells from CB HSCs *in vitro* an attractive candidate for NK cell immunotherapy.

## NK Cell Activation

**Figure 1:**
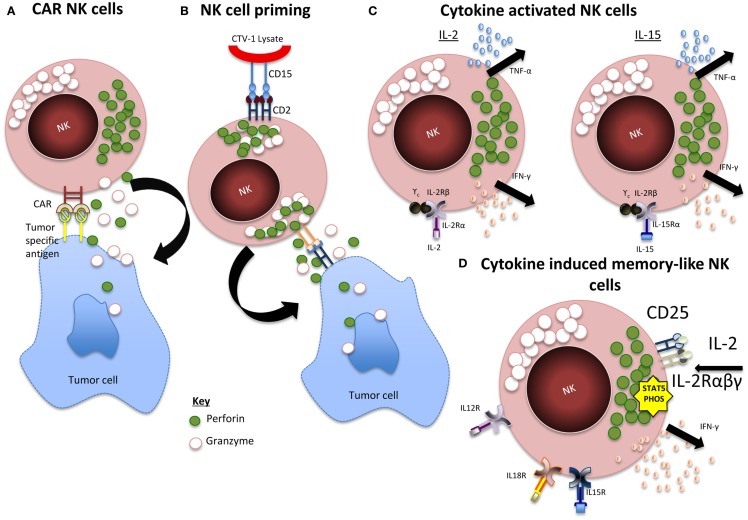
**Summary of NK activation mechanisms**. **(A)** CAR NK cells. Expression of chimeric antigen receptor (CAR) specific for tumor-associated cell surface antigens redirects NK cells to malignant cells and facilitates cytotoxic activity. **(B)** Primed NK cells. Engagement of CD2 within CD15 of CTV-1 ligand leads to granule polarization and NK cell function is triggered by the engagement of at least one more activating receptor from a tumor cell. **(C)** Cytokine activated NK cells. IL-2 and IL-15 activation leads to activation of JAK/STAT, PI3K, MAPK, and NF-κB pathways. **(D)** CIML NK cells. IL-12, IL-15, and IL-18 induces a rapid and prolonged expression of CD25, resulting in a highly functional high-affinity IL-2 receptor. The receptor responds to picomolar concentrations of IL-2 leading to STAT5 phosphorylation and release of IFN-γ.

As reviewed in Table 1, there have been many studies that well document the expansion of NK cells *in vitro*, however, we are yet to obtain a clinically successful product, which proliferates and persists *in vivo* inducing consistent efficacy. This could be because we are yet to identify the optimum activation method and status of the cells before infusion. As seen in Figure 1, whether the cells should be incubated with cytokines, genetically engineered, differentiated into a “memory-like” phenotype, or primed using NK non-susceptible cell lines are all options that need to be considered.

**Table 1:** **NK cells in the clinic: trials so far**.

Initialpopulation	Feeder cells	Fold expansion *in vitro* (purity)	*In vitro* cytokine admin	Condition	Treatment and *in vivo* cytokine admin	Dose	*In vivo* expansion	Clinical outcome	Reference
**AUTOLOGOUS**									
CD3 depleted PBMCs	LCL cell line (LAZ388)	43 ± 26 in 13–21 days (90%)	IL-2	MRC	High dose IL-2 + LANAK following initial PR to IL-2 alone	N/A	N/D	Induced clinical response 15–30% patients	(38)
PBMCs	None	No expansion	IL-2	Advanced CRC and NSCLC	Multiple infusions of NK cells + IL-2 + Hsp70 peptide TKD	1–7.5 × 10^6^/kg	Multi-infusion trial	Well tolerated and safe, no significant tumor response	(39)
PBMCs	Wilms tumor cell line (HFWT)	113 in 14 days (96%)	IL-2	Malignant glioma	Multiple infusions + IFN-β	N/A	Multi-infusion trial	Well tolerated, no toxicity, 3 PR, 2 MR, 4 NC, and 7 PD	(12)
PBMCs	αGalCer-pulsed autologous PBMCs	10^1^–10^3^ 21 days (70% viability)	IL-2	Recurrent or advanced NSCLC	Infusion of *ex vivo* expanded Vα24NKT cells	5 × 10^7^/m^2^	Reduced function in some patients	Well tolerated, no toxicity	(40)
CD3^−^/CD56^+^ PBMCs	EBV-LCL (TM-LCL)	53–683 in 14 days (99.7%)	IL-2	CLL and metastatic tumors	Infusion of NKs + IL-2 after PEN/BOR	1 × 10^8^/kg	Multi-infusion trial	Well tolerated and some pre-clinical evidence of anti-tumor response	(41)
CD3^−^ PBMCs	Auto PBMCs	278–1097 in 21–26 days (91–97%)	IL-2	Metastatic melanoma and RCC	Infusion of activated NKs + IL-2 after CY/FLU regimen	1.88–7.6 × 10^10^/kg	NK persistence 7 days post infusion	No toxicity or clinical response	(42)
**AUTOLOGOUS/ALLOGENEIC**									
CD56^+^/CD3^−^ PBMCs	4-1BBL^+^IL-15Rα^+^ aAPCs	12–160 in 7–9 days (68–99%)	IL-2	MM	Infusion of NKs + IL-2 after BOR/CY/FLU	2 × 10^7^–2 × 10^8^/kg	Significant *in vivo* expansion fresh product	Well tolerated, no toxicity	(43)
**ALLOGENEIC**									
CD3^−^/CD56^+^ PBMCs	N/A	No expansion	None	High risk myeloid malignancies	Infusion of NKs post haplo-HSCT	0.21–1.41 × 10^7^/kg	N/D	Well tolerated, increased donor chimerism in 2/5 patients	(19)
CD3^−^/CD56^+^ PBMCs	None	5 in 12 days (95%)	IL-2	Multiple relapse ALL and AML	Repeat infusions of activated NKs post-HSCT	8.9–29.5 × 10^6^/kg	N/D	Well tolerated, no toxicity	(44)
CD3^−^ PBMCs	None	No expansion	None	Metastatic melanoma, RCC, refractory Hodgkin’s, and AML	Infusion of NKs + IL-2 after Lo-CY/mPred, FLU, or Hi-CY/FLU	1 × 10^5^–2 × 10^7^/kg	*In vivo* NK expansion in Hi-Cy/Flu patients	CR in 5/19 poor prognosis patients	(10)
CD3^−^ PBMCs	None	No expansion	IL-2	Myeloma	Infusion of activated NKs + IL-2 after FLU/MEL regimen and auto-PBSCT	1.7 × 10^6^/kg	Donor cells persisted and lost by day 9–14	CR in 50% patients	(45)
PBMCs	None	1036 in 19 days (88% viability)	OKT3 and IL-2	CRC, carcinoma and B-CLL	Infusion of activated NKs + IL-2 after haplo-HSCT	8.1–40.3 × 10^6^/kg	Multi-infusion trial	Minor response in 2 patients	(46)
CD3^−^/CD56^+^ PBMCs	None	No expansion	None	AML	Infusion of NKs + IL-2 after CY/FLU regimen	0.5–8.1 × 10^7^/kg	Significant *in vivo* expansion observed at day 14 (5800/mL)	100% EFS at 2 years	(47)
CD3^−^ PBMCs	None	No expansion (43 ± 11%)	None	Lymphoma	Infusion of NKs + IL-2 after RTX/CY/FLU	0.2–40 × 10^7^/kg	NK cells not detected 7 days post infusion	2 CR/2PR	(48)
CD3^−^/CD56^+^ PBMCs	None	32–131.3 in 20–23 days (82.7–99.6%)	HC and IL-15	Advanced NSCLC	Infusion of pre-activated NKs	0.2–29 × 10^6^/kg	Multi-infusion trial	PR in 2 patients best response with most infusions	(49)
CD56^+^ selected PBMCs	None	No expansion	None	AML	Infusion of NKs + IL-2 after CY/FLU regimen	1.11–5.0 × 10^6^/kg	Donor NKs detected up to 17 days post first infusion	CR 6/13 patient	(50)
CD3^−^/CD56^+^ PBMCs	None	No expansion	IL-2 for half of patients	AML, ALL, neuroblastoma, and RMS	Multiple infusions of pre-activated and resting NKs after haplo-HSCT	6–45.1 × 10^6^/kg	NK cells detected at 24 h	Two patients with neuroblastoma alive at 2 years	(51)
CD3^−^ PBMCs	None	No expansion (70% viability)	IL-2	Breast and ovarian carcinoma	Infusion of pre-activated NKs + IL-2 after CY/FLU with/without TBI	8.33 × 10^6^–3.94 × 10^7^/kg	No eligible patients met predefined criterion for successful *in vivo* expansion	TBI improved longevity of NK engraftment	(52)
CD56^+^/CD3^−^ PBMCs	None	No expansion	None	Leukemia and malignant solid tumors	Multiple NK infusions after ATG/OKT3 and hapol-HSCT	0.3–3.8 × 10^7^/kg	N/D	No significant clinical response	(53)
CD56^+^/CD3/CD19^−^ PBMCs	None	No expansion (53%)	IL-2	Relapsed/primary AML	Infusion pre-activated NKs after IL-2DT	2.6 ± 1.5 × 10^7^/kg	*In vivo* expansion enhanced with T-REG depletion	Well tolerated, no toxicity	(11)
CD56^+^CD3^−^ PBMCs	4-1BBL^+^IL-15Rα^+^ aAPCs	9–11 days (>90%)	IL-15	EWS, DSRCT, and RMS	CY/FLU/MEL/G-CSF	1 × 10^5^/kg	Multi-infusion trial	5/9 patients experienced acute GVHD	(25)
**CELL LINES**									
NK92 cell line	None	>200 in 15–17 days	IL-2	RCC and malignant melanoma	Infusion of *ex vivo* expanded NK-92 cells	Up to 3 × 10^9^/m^2^	Multi-infusion trial	Well-tolerated possible response in 2 patients	(27)
NK92 cell line	None	2 in 32 h	IL-2	Solid tumors, sarcomas, leukemias, and lymphoma	Infusion of *ex vivo* expanded NK-92 cells	Up to 1 × 10^10^/m^2^	Persist in circulation up to 48 h	Well-tolerated possible response in lung cancer patients	(28)
CD56^+^CD3^−^ PBMCs	None	5 in 14 days (>95%)	IL-2	Multiple relapsed neuroblastoma	Infusion pre-activated NK cells	7.8–45.1 × 10^6^/kg	No clear expansion	No toxicity observed	(54)

*ALL, acute lymphoblastic lymphoma; AML, acute myeloid leukemia; ATG, anti-thymocyte globulin; B-CLL, B cell chronic lymphocyte leukemia; BOR, bortezomib; CLL, chronic lymphocyte leukemia; CR, complete response; CRC, colorectal carcinoma; CY, cyclophosphamide; DSRCT, desmoplastic small round cell tumor; EWS, Ewing sarcoma; FLU, fludarabine; G-CSF, granulocyte colony stimulating factor; HC, hydrocortisone; MEL, melphalan; MM, multiple myeloma; mPred, methylprednisolone; MRC, metastatic renal carcinoma; N/D, not determined; NSCLC, non-small cell lung carcinoma; PEN, pentostatin; PR, partial response; RCC, renal cell carcinoma; RMS, rhabdomyosarcoma; RTX, rituximab; TBI, total body irradiation*.

Cytokine activation has always been a popular method of stimulating NK cells as it is a well-documented pathway of activation *in vivo* and different cytokines can give rise to the same signaling patterns while differing in their effects on development, activation, and proliferation. IL-2 stimulates cellular proliferation and enhances cytotoxicity, however, it has been noted that this only affects a small sub-population for an extended period (55). IL-15 significantly improves NK cell survival although it only stimulates minimal expansion (56). Furthermore, the toxic effects of the *in vivo* administration of cytokines cannot be ignored, IL-2 risks vascular leak syndrome caused by the stimulation of endothelial cells through the IL-2 receptor (57) and preferentially expands T regulatory cells, which mediate immune suppression (58). Studies with IL-15 in non-human primates have only shown transient toxicity; however, its reduced half life may suggest the need for more frequent dosing in therapeutic applications (59).

A method to avoid such life threatening conditions through *in vivo* administration of cytokines is expanding or stimulating the cells *in vitro*. NK cells have always been considered a member of the innate immune system incapable of producing memory. However, in 2006, it was first observed that NK cells could mediate a long-lived antigen-specific adaptive response independently of other lymphocytes (60). Sun and colleagues (61) later identified an immunological memory in NK cells from MCMV infected mice and it was demonstrated that NK cells pre-activated with IL-12 and IL-18 infused into a naïve host and later re-stimulated showed enhanced IFN-y production (62). This model was later transferred to an *in vitro* model stimulating human cells showing the same results (63). This improvement in cytokine production offers the potential for enhanced GvL effect and a clinical trial is currently in progress targeting relapsed and refractory AML (NCT01898793).

It has been reported that resting NK cells require a two-stage activation process known as “priming” and “triggering” (64). This states that tumors resistant to NK cell killing evade lysis by failing to prime the cell; however, Mark Lowdells group were able to identify a cell line, which could prime the cell without triggering cytokine production or cytolytic activity. This led to the development of an NK cell priming technique that readied the cells for killing, which was still maintained post cryopreservation (65). Primed NK cells from patients with multiple myeloma have also been proven to kill NK cell resistant malignant plasma cells (66). Preliminary data from an ongoing transitional phase I/II clinical trial showed that without cytokine administration primed NK cells from HLA haploidentical-related donors can persist *in vivo* with no toxic effects (67).

## Genetic Engineering

Although currently restricted to pre-clinical models the use of chimeric antigen receptor (CAR)-expressing NK cells has the potential to offer enhanced effector cell function of increased specificity. Anti-CD19 CAR T cells have effectively demonstrated their ability to induce long-term remission in patients with B cell malignancies (68). However, concern associated with CAR T cell therapy extends to GvHD, on target/off tumor effects, and tumor lysis syndrome. By contrast, allogeneic CAR-engineered NK cells are expected to induce anti-tumor effects and dissipate after a few days (23). As previously reviewed by Glienke et al. in 2015, current work in the field has focused mainly on targeting CD19 and CD20, however, CARs, which target CS1 and CD138 for multiple myeloma, GD2 and CD244 for neuroblastoma, HER-2 for epithelial carcinomas, and GPA7 for melanoma, are also beginning to indicate promising results.

## Immune Escape Mechanisms

Not all tumors are susceptible to NK cell mediated killing, as some cancer cells have developed the ability to escape detection by the immune system. Mechanisms that regulate the evasion of tumor cells by NK cells extends to the down-regulation of activating receptor ligands for NKG2D (69), the production of soluble stress-induced ligands, such as MICA, which degrades NKG2D leading to NK cell inhibition (70) and the release of suppressive cytokines such as IL-10 and TGF-β (71). Some success has been seen by NK cell immunotherapy targeting hematological malignancies; however, this has not been transferred to solid tumors. This could be a result of the increased concentration of immunosuppressive cytokines and ligands around a tumor mass, method to overcome such escape mechanisms could provide further potential for NK cells to not only target hematological malignancies but also solid tumors.

## Concluding Remarks

Natural killer cell immunotherapy has been a promising option for providing specialized and target specific treatment for a therapy in its own right and as a supportive one in infection or transplantation. Although some mechanisms of NK cell biology are yet to be elucidated as we make progress in the field an effective clinical NK cell immunotherapy will become more achievable. A standard clinical regimen is still to be elucidated and obstacles such as cell dose, activation status, method of expansion, drug complement, and source are still to be determined.

It has always been thought that high numbers of NK cells are necessary for a successful clinical product. However, numerous groups have managed to successfully generate high numbers of functional NK cells *in vitro* although the lack of clinical effect and significant cost implications cannot be ignored. The high-cell number requirement is likely to be the result of a “success in numbers” approach with there being a significant loss of cells through *in vivo* targeting and just a small sub-population of effector cells that will target the tumor. Perhaps work should therefore be refocused on the infusion of a small population of cells with optimum pre-activation status that will traffic to the tumor site and would not be suppressed by tumor evasion mechanisms. This is a significant goal to achieve considering the variety of NK cell populations occurring naturally in the body. However, the absence of a labor-intensive long-term culture system would mean this method would pose significantly reduced cost implications. Once techniques have been optimized and streamlined there would therefore be a greater possibility of NK cell immunotherapy being routinely adopted as a clinical therapy in the future.

## Conflict of Interest Statement

The authors declare that the research was conducted in the absence of any commercial or financial relationships that could be construed as a potential conflict of interest.
